# EBV-Driven Lymphoproliferative Disorders and Lymphomas of the Gastrointestinal Tract: A Spectrum of Entities with a Common Denominator (Part 3)

**DOI:** 10.3390/cancers13236021

**Published:** 2021-11-30

**Authors:** Magda Zanelli, Francesca Sanguedolce, Andrea Palicelli, Maurizio Zizzo, Giovanni Martino, Cecilia Caprera, Valentina Fragliasso, Alessandra Soriano, Fabrizio Gozzi, Luca Cimino, Francesco Masia, Marina Moretti, Moira Foroni, Loredana De Marco, David Pellegrini, Hendrik De Raeve, Stefano Ricci, Ione Tamagnini, Alessandro Tafuni, Alberto Cavazza, Francesco Merli, Stefano A. Pileri, Stefano Ascani

**Affiliations:** 1 Pathology Unit, Azienda USL-IRCCS di Reggio Emilia, 42123 Reggio Emilia, Italy; andrea.palicelli@ausl.re.it (A.P.); moira.foroni@ausl.re.it (M.F.); loredana.demarco@ausl.re.it (L.D.M.); stefano.ricci@ausl.re.it (S.R.); ione.tamagnini@ausl.re.it (I.T.); alberto.cavazza@ausl.re.it (A.C.); 2 Pathology Unit, Policlinico Riuniti, University of Foggia, 71122 Foggia, Italy; francesca.sanguedolce@unifg.it; 3 Surgical Oncology Unit, Azienda USL-IRCCS di Reggio Emilia, 42123 Reggio Emilia, Italy; maurizio.zizzo@ausl.re.it; 4 Pathology Unit, Azienda Ospedaliera Santa Maria di Terni, University of Perugia, 05100 Terni, Italy; gio.martino@gmail.com (G.M.); ceciliacaprera@libero.it (C.C.); d.pellegrini@aospterni.it (D.P.); s.ascani@aospterni.it (S.A.); 5 Laboratory of Translational Research, Azienda USL-IRCCS di Reggio Emilia, 42123 Reggio Emilia, Italy; valentina.fragliasso@ausl.re.it; 6 Gastroenterology Division, Azienda USL-IRCCS di Reggio Emilia, 42123 Reggio Emilia, Italy; alessandra.soriano@ausl.re.it; 7 Ocular Immunology Unit, Azienda USL-IRCCS di Reggio Emilia, 42123 Reggio Emilia, Italy; fabrizio.gozzi@ausl.re.it (F.G.); luca.cimino@ausl.re.it (L.C.); 8 Dipartimento di Medicina, Università degli Studi di Perugia, 05100 Terni, Italy; francesco.masia@unipg.it (F.M.); marina.moretti@unipg.it (M.M.); 9 Pathology, University Hospital Brussels, 1090 Brussels, Belgium; hendrik.de.raeve@olvz-aalst.be; 10 Pathology, O.L.V. Hospital Aalst, 9300 Aalst, Belgium; 11 Pathology Unit, Department of Medicine and Surgery, University of Parma, 43121 Parma, Italy; alessandro.tafuni@unipr.it; 12 Hematology Unit, Azienda USL-IRCCS di Reggio Emilia, 42123 Reggio Emilia, Italy; francesco.merli@ausl.re.it; 13 Haematopathology Division, European Institute of Oncology-IEO IRCCS Milan, 20141 Milan, Italy; stefano.pileri@unibo.it

**Keywords:** Epstein–Barr virus, chronic active EBV infection, extranodal NK/T-cell lymphoma, nasal type, post-transplant lymphoproliferative disorders

## Abstract

**Simple Summary:**

The Epstein–Barr virus (EBV) is a commonly occurring virus, infecting more than 90% of the world population, often early in life. However, only a minority of individuals develop EBV-driven diseases at some point in their lifetime. EBV is associated with several neoplasms including epithelial, mesenchymal and lymphoid tumors. EBV-driven lymphoid proliferations encompass a wide spectrum of diseases with different biological behaviors, developing frequently, although not always, in conditions of immunosuppression. The diagnosis is often complicated and requires a strict combination of clinical, pathological and molecular findings. The aim of this review, divided into three parts, is to provide an update on EBV-driven lymphoproliferative disorders arising in the gastrointestinal tract. In this review, we discuss the chronic active EBV infection of T-cell and NK-cell type, its systemic form; extranodal NK/T-cell lymphoma, nasal type and post-transplant lymphoproliferative disorders.

**Abstract:**

EBV is the first known oncogenic virus involved in the development of several tumors. The majority of the global population are infected with the virus early in life and the virus persists throughout life, in a latent stage, and usually within B lymphocytes. Despite the worldwide diffusion of EBV infection, EBV-associated diseases develop in only in a small subset of individuals often when conditions of immunosuppression disrupt the balance between the infection and host immune system. EBV-driven lymphoid proliferations are either of B-cell or T/NK-cell origin, and range from disorders with an indolent behavior to aggressive lymphomas. In this review, which is divided in three parts, we provide an update of EBV-associated lymphoid disorders developing in the gastrointestinal tract, often representing a challenging diagnostic and therapeutic issue. Our aim is to provide a practical diagnostic approach to clinicians and pathologists who face this complex spectrum of disorders in their daily practice. In this part of the review, the chronic active EBV infection of T-cell and NK-cell type, its systemic form; extranodal NK/T-cell lymphoma, nasal type and post-transplant lymphoproliferative disorders are discussed.

## 1. Introduction

EBV is a gamma herpesvirus that preferentially infects B lymphocytes via the CD21 cell surface protein [1,2,3,4,5,6,7,8,9,10,11]. Once EBV infection occurs, the virus cannot be eradicated, persisting in a latent phase within B cells throughout life.

EBV has been demonstrated to contribute to the development of different types of tumors. Epithelial cells, mesenchymal cells and lymphocytes of B, T, and NK-cell origin may be infected by EBV. Although T-lymphocytes and NK-cells may be the target of virus, the specific receptor leading to EBV infection in these cell types remains unknown [12,13,14,15].

Under conditions of immunosuppression (IS), altering the balance between the virus and the host immune response, infected cells may proliferate, causing different types of tumors, including a wide range of EBV-related lymphoproliferative disorders (LPDs) [11].

In western countries, B lymphocytes are the predominantly infected cells, often leading to LPDs of B-cell origin, whereas in Asia and central/south America, T- and NK-cells are more often infected.

EBV-linked LPDs may occur not only under circumstances of IS. Apparently immunocompetent individuals may develop a chronic disease characterized by prolonged infectious mononucleosis (IM)-like symptoms and a high EBV DNA load in the peripheral blood (PB), which is called a chronic active EBV (CAEBV) infection, mainly of T/NK-cell type and, in a minority of cases (2%), of B-cell type [8,12,13,14,15].

Our review summarizes the current knowledge on EBV-associated LPDs involving the gastrointestinal tract (GIT) with the aim of increasing our awareness on this heterogeneous group of diseases that often present overlapping clinical and morphological features, despite different biological behavior, hence, deserving the attention of both pathologists and clinicians.

The review consists of three parts. In part 1 and 2 we analyzed the clinicopathologic features of LPDs of B-cell origin such as an Epstein–Barr virus mucocutaneous ulcer (EBVMCU) [8,16,17,18,19,20,21,22,23,24,25,26,27,28,29,30,31,32,33], EBV-positive diffuse large B-cell lymphoma not otherwise specified (EBV-positive DLBCL, NOS) [8,34,35,36,37,38,39,40,41,42], classic Hodgkin lymphoma (cHL) [8,43,44,45,46,47,48,49,50,51,52,53,54,55], plasmablastic lymphoma (PBL) [8,56,57,58,59,60,61,62,63,64,65,66,67,68,69,70,71,72,73,74], extra-cavitary primary effusion lymphoma (EC-PEL) [8,75,76,77,78,79,80,81,82,83,84,85,86,87,88,89,90,91,92], and a Burkitt lymphoma (BL) [8,93,94,95,96,97,98,99,100,101,102,103,104,105,106,107,108].

In this part of the review, we discuss the CAEBV infection of T-cell and NK-cell type, its systemic form, extranodal NK/T-cell lymphoma, nasal type (ENKTL-NT) and EBV-associated post-transplant lymphoproliferative disorders (PTLDs).

## 2. CAEBV of T-Cell and NK-Cell Type, Systemic Form

### 2.1. General Features and Etiology

CAEBV was originally referred to as chronic IM and is characterized by persistent (IM)-like symptoms after acute EBV infection [8,12,13,14,15,109,110,111,112,113,114,115,116,117,118,119,120].

In primary EBV infections, presenting as IM, symptoms such as fever and lymphadenopathy usually resolve within a few weeks, although they may persist even longer and the level of EBV DNA in PB usually remains high for the first month of disease.

Infrequently, patients are unable to control EBV infection and, as a result, develop a chronic course of the disease. This course of the disease is referred to as the CAEBV disease [8,109,110,111,112,113,114,115,116].

Initially, the required duration of symptoms for the classification of CAEBV was more than six months [111].

Currently, according to the 2017 WHO classification, the diagnostic criteria for CAEBV of T-cell and NK-cell type, systemic form are as follows: (1) IM-like symptoms lasting more than three months; (2) increased EBV DNA (>10^2.5^ copies/mg) in PB; (3) histological evidence of organ disease; (4) occurrence of EBV RNA or viral protein in lymphocytes of involved tissues.

The guidelines define CAEBV of T-cell and NK-cell type, and its systemic form as a disease distinct from known conditions of immunosuppression (IS). The disease affects immunocompetent hosts, without apparent causes of IS, tumors or autoimmune diseases [8,109,110,111,112,113,114,115,116].

In the current WHO classification, CAEBV is categorized among EBV-positive T-cell and NK-cell lymphoproliferative diseases of childhood and is defined as a polyclonal, oligoclonal or frequently monoclonal LPD. Most CAEBV cases are reported in Asia (Japan, Korea, China and Taiwan), although the disease is even observed in individuals from Latin America [8]. In Western countries, the incidence is much lower and, unlike CAEBV in Asia, where EBV is detected in T-cells or NK-cells, in Western countries, EBV is detected in B lymphocytes [8,112]. Genetic polymorphism in genes related to EBV immune response has been hypothesized for the strong racial predisposition of CAEBV [8]. An impairment of EBV-specific cytotoxic T-lymphocytes activity is found in CAEBV patients [8].

The disease affects children and adolescents more frequently, although it is reported in adults [8,113]. Persistent fever, hepatosplenomegaly and lymphadenopathy represent the IM-like symptoms occurring in 50% of patients. EBV-infected cells may infiltrate almost any organ, resulting in organ failure.

The main clinical feature of the disease is inflammation, whereas solid masses are rarely detected. Common manifestations include severe mosquito bite allergy (SMBA), skin rash, hydroa vacciniforme (HV)-like eruptions, diarrhea and uveitis. However, as almost any organ may be involved, the clinical presentation is variable, and often delays the correct diagnosis. The clinical course varies from a prolonged course even of years to a rapid and aggressive course. Patients with NK-cell disease show skin rash, hypersensitivity to mosquito bites and mild systemic symptoms with a prolonged course, whereas in patients whose T-cells are predominantly infected, systemic symptoms are severe with rapid disease progression [8,114]. Severe and often fatal complications such as hemophagocytic lymphohistiocytosis (HLH), multi-organ failure and progression to overt leukemia/lymphoma may occur [8,115,116]. Approximately 16% of CAEBV patients develop NK/T-cell lymphoma or aggressive NK-cell leukemia [8,116].

Among major laboratory findings, there are signs of liver dysfunction and pancytopenia. Elevated levels of IgG and even IgA antibodies against EBV viral capsid antigen (VCA) or early antigen (EA) are often detected. By definition, high levels of EBV DNA in PB are always present and more specific than high titres of EBV antibodies. NK-cell CAEBV shows high levels of IgE and often lower titres of anti-EBV antibodies compared with T-cell CAEBV cases which often displays high levels of EBV-specific antibodies.

### 2.2. CAEBV and GIT

The involvement of GIT in CAEBV is rare, with only a few cases reported so far, and is extremely difficult to distinguish from inflammatory bowel disease (IBD) [117,118,119,120].

Patients are often young adults [118,119,120]. GI symptoms include vomiting, diarrhea, hematochezia and abdominal pain, which may be associated with extra-gastrointestinal manifestations. Acute or intermittent fever is observed in almost all patients with GI CAEBV, whereas hepatomegaly, splenomegaly and lymphadenopathy are observed in approximately half the cases.

Laboratory findings include extremely high levels of ferritin associated with increased levels of inflammatory markers such as C-reactive protein (CRP) and the erythrocyte sedimentation rate (ESR). Increased EBV DNA (>10^2.5^ copies/mg) is always detected in PB.

Endoscopy shows single or multiple ulcers mainly located in the colon and, in decreasing order of frequency, in the small intestine and stomach. Radiology may identify a thickening of the gastric or intestinal wall and/or enlarged mesenteric lymph nodes [120].

In GI CAEBV, the histological features are rather subtle and easily overlooked, unless evaluated in combination with the clinical history and laboratory data.

### 2.3. Histology, Immunophenotype and Genetic Profile

The main difficulty in diagnosing CAEBV is in the lack of changes observed, suggestive of malignancy in the affected tissues.

CAEBV histology often resembles nonspecific inflammatory changes; hence, it is essential to consider the whole clinical history and, in cases of sustained inflammation of unknown origin, CAEBV should always be taken into consideration. The identification of EBV infection by in situ hybridization for EBV-encoded RNA (EBER) in affected tissues is required to confirm the diagnosis. Lymph node histology often shows nonspecific features, including follicular hyperplasia, paracortical hyperplasia, epitheliod granulomas, focal necrosis and polymorphic infiltrate in the interfollicular zones. Red-pulp congestion and white-pulp atrophy are common, but otherwise nonspecific, findings observed in the spleen. Depending on the affected organs, CAEBV mimics viral hepatitis, viral myocarditis, interstitial pneumonia or dermatitis. Bone marrow (BM) is usually unremarkable. If HLH complicates CAEBV, features of hemophagocytosis may be observed particularly in BM, liver and lymph nodes.

In GI CAEBV, morphologic changes are often subtle. Ischemic features and erosions are frequently observed, whereas crypt abscesses are usually absent [120]. The infiltrate involving the mucosa and occasionally the submucosa and muscle layers consists of small to medium-sized lymphocytes either lacking atypia or with a mild to moderate degree of atypia. Lymphoid aggregates are observed in some cases and lymphocytes may show increased cytoplasm compared with mature small lymphocytes [120]. Focal necrosis may also be present.

EBER is the gold standard to demonstrate EBV infection, although the percentage of EBER-positive cells required for the definition of EBV infection is not well defined. Some studies suggest a requirement of at least 30 EBER positive cells/high power fields (HPF) in biopsy samples and more than 100 EBER positive cells/HPF in surgical specimens [118,119,120].

EBER-positive cells are predominantly of T-cell origin in 59% of cases, mostly with a CD4 phenotype, although CD8-positive CAEBV cases are reported [120]; in 41% of cases, NK cells are infected, whereas in a low percentage of CAEBV cases (4%) both T- and NK-cells are affected. B-cells are rarely involved (2–3%) although usually in the Western population [112]. EBV-infected cells express EBNA1, LMP1 and LMP2A, indicating a latency pattern type 2.

T-cell receptor (TCR) gene rearrangement indicates monoclonality in the majority of CAEBV cases (84%), oligoclonality in 11% and polyclonality in only 5% of cases [121].

Based on the cytology and clonality of the lymphoid cells, the current WHO classification adopts a subdivision into different categories. Polymorphic and polyclonal proliferation are classified as A1; A2 is polymorphic and monoclonal and A3 is monomorphic and monoclonal. This classification reflects the spectrum of diseases under the umbrella term of CAEBV, from LPDs (A1–A2) to frank lymphomas (A3). The B category includes monomorphic and monoclonal cases with a fulminant course, equivalent to systemic EBV-positive T-cell lymphoma of childhood [8,122].

### 2.4. Differential Diagnosis

In patients suffering from a sustained inflammation of unknown origin, CAEBV should be considered. Clinicians’ awareness of this rare disease is indeed the first diagnostic step to recognize CAEBV. Then, PB tests such as anti-VCA-IgG, anti-EA-IgG, anti-VCA-IgA, anti-EA-IgA together with quantification of EBV DNA load in PB should be performed. The acute phase of primary infection, that is, IM, should be excluded, checking the patient’s clinical history. Anti-VCA IgM are usually present in acute IM, unlike in CAEBV.

In the absence of the clinical suspicion of CAEBV, a histological diagnosis is practically impossible as CAEBV mimics nonspecific inflammatory processes.

GI CAEBV needs to be differentiated from IBD, ENKTL-NT and EBVMCU.

A differential diagnosis from IBD can be challenging, especially considering that there are cases of IBD superimposed with EBV infection. If CAEBV is limited to the GIT, and lacks more characteristic features such as lymphadenopathy, splenomegaly and hepatomegaly, differentiating IBD from CAEBV can be very difficult. GI symptoms and increased levels of inflammatory markers (ESR and CRP) are present in both diseases. However, useful clues more in favor of CAEBV are intermittent high fever and extremely high level of ferritin [118]. Endoscopically, the cobblestone appearance observed in Crohn’s disease and the diffuse and continuous GI involvement typical of ulcerative colitis are absent in CAEBV. EBV DNA in PB may be positive even in IBD patients, although usually with lower values compared to CAEBV. EBER-positive cells may be found in IBD samples; therefore, the presence of EBER-positive cells alone is not enough to diagnose CAEBV. A high index of suspicion is required if the clinical history and laboratory data are consistent with CAEBV.

Unlike CAEBV, ENKTL-NT is classically localized in the upper aerodigestive tract and then, in the course of thr disease, disseminates to other sites including the GIT. In some cases, ENKTL-NT may occur primarily in the GIT, usually presenting with perforation or GI bleeding. The angiocentric and angiodestructive pattern of growth of the lymphoid infiltrate associated with coagulative necrosis is an helpful clue in the differential with CAEBV. The main lymphoproliferative disorders of T/NK-cell origin involving the GIT are summarized in Table 1.

EBVMCU is a B-cell LPD presenting with isolated ulcers in the oropharynx, skin and GIT [8]. Unlike CAEBV, which usually occurs in young adults without known immunodeficiency, EBVMCU often occurs in elderly patients or in individuals with iatrogenic IS. In EBVMCU, the ulcers frequently consist of a polymorphic infiltrate containing EBV-positive atypical cells, often resembling Hodgkin cells, with strong CD30 positivity and a variable expression of B cell markers. The disease course is completely different, as EBVMCU is an indolent disorder often regressing spontaneously or upon removal of the cause of IS, whereas CAEBV prognosis is often dismal.

### 2.5. Treatment and Outcome

The behavior of the disease is variable, depending on the EBV viral load, the host immunity and the type of the infected cells. Patients with NK-cell infection show a more favorable course with mild systemic symptoms and a 5-year survival rate of 87% compared with T-cell CAEBV patients, who experience a more aggressive course with a survival rate of 59% [8,113]. CAEBV patients with adult onset of disease show a worse outcome [8,113]. If complications such as HLH or lymphoma develop, CAEBV can be fatal. Therefore, treatment must begin before these serious complications occur.

To date, the only known effective cure is an allogenic hematopoietic stem cell transplant (allo-HSCT). Fifteen-year overall survival (OS) is 60.6% in patients treated with allo-HSCT compared to 25.7% in patients without allo-HSCT [121]. In addition, the OS was significantly higher (85%) in patients receiving reduced intensity conditioning (RIC) compared to patients with myeloablative conditioning (54.5%), indicating that the positive effect of allo-HSCT depends mainly on the reconstruction of the hematopoietic and immune system, rather than on the antineoplastic effect of chemotherapy (CT) and radiotherapy (RT) [123,124]. The outcome of CAEBV after allo-HSCT depends on the activity of the disease as patients with an active CAEBV characterized by fever, liver dysfunction, vasculitis or progressive skin lesions have a poor prognosis after allo-HSCT [121]. Poor results have been obtained with different types of CT with the aim of reducing disease activity [125].

## 3. ENKTL-NT

### 3.1. General Features and Etiology

ENKTL-NT is an aggressive, angiocentric and angiodestructive lymphoma principally affecting the nasal or upper aerodigestive tract and, more rarely, non-nasal sites.

The disease is prevalent in Asia (Japan, Korea, Taiwan, China, Hong Kong and Thailand) and Latin America (Mexico, Guatemala, Brazil) [8,126,127,128,129,130,131,132,133,134,135]; it more often affects adult males with a median age of 40–60 years, although occurrence in children is rarely reported.

EBV infection and genetic predisposition are likely to be involved in the pathogenesis, thereby explaining the characteristic ethnic distribution of this lymphoma [8,126,127,128]. The way EBV contributes to the genesis of the disease is still not completely clarified; possible EBV-related oncogenic mechanisms may be the overexpression of the oncogenic protein LMP1 as well as the activation of the NF-kB signaling pathway [127,128].

Diverse genetic abnormalities are likely to contribute to ENKTL-NT tumorigenesis. Deletion of chromosomal region at 6q21-23, containing multiple tumor suppressor genes (*PRDM1*, *PTPRK*, *FOXO3*) is frequently found in ENKTL-NT (36–60% of cases); additionally, genetic alterations of the *JAK-STAT* pathway and mutations involving tumor suppressor genes such as *BCOR*, *DDX3X* and *TP53* have been identified [126,129,130,131].

Clinical manifestations vary according to the primary site of disease occurrence. The most common clinical symptoms are nasal obstruction with epistaxis, followed by necrotic lesions involving the upper aerodigestive tract and midface with an extension to contiguous areas and massive midfacial destruction [8,126]. Systemic symptoms such as fever and weight loss are variably present.

At diagnosis, patients usually have localized disease (stage I or II), although, during the clinical course, the involvement of other sites such as GIT, skin, lymph nodes and the central nervous system (CNS) is common. BM involvement is rare at presentation, but may occur in the clinical course. HLH is a serious complication of this lymphoma, which may develop especially in cases with BM involvement. In approximately 20–30% of cases, the disease may occur in extranasal sites such as the skin, soft tissue, testis, salivary gland, liver and GIT, causing organ-specific manifestations [8,132,133,134,135]. Although secondary nodal involvement may rarely occur, primary nodal presentation is considered very rare [136] and these cases should be better classified as a variant of peripheral T-cell lymphoma [8].

Cases occurring outside the nasal region seem to have a worse outcome than the classic nasal form and often present at advanced stages [132,133,134,135].

### 3.2. ENKTL-NT and GIT

Primary GI ENKTL-NT is rare, representing 2.7% of ENKTL-NT [137,138]. Clinical manifestations include abdominal pain, GI bleeding, intestinal obstruction and bowel perforation, often leading to surgery.

The small intestine is most often involved, especially the ileum (29%) and jejunum (17%) followed by duodenum (4%). The large bowel represents the second most common site with the right colon more frequently involved than the distal tract (cecum 14%; ascending colon 11%; transverse colon 9%; descending colon 4%; rectosigmoid colon 6%); whereas the stomach (5%) and the esophagus (1%) are more rarely affected.

A discrete proportion of patients may present multiple involvement of GIT such as concomitant small and large bowel involvement. Other extranodal sites such as the nasal tract may be concomitantly involved; the majority of patients present B symptoms and the disease is often at an advanced stage at presentation.

### 3.3. Histology, Immunophenotype and Genetic Profile

ENKTL-NT usually presents as a mucosal ulcer or an ulcerated mass. Histologically, the neoplasm consists of a diffuse infiltrate of variably sized lymphoid cells, although small to medium-sized cells, often with clear cytoplasm and irregularly folded nuclei, usually predominate. Cells of large size may be present. The neoplastic elements are generally accompanied by an abundant inflammatory component and necrosis.

An angiocentric and angiodestructive growth pattern is commonly present, although it is worth mentioning that these characteristic features may not be evident in small biopsy samples [135].

The disease is often of NK-cell origin and the neoplastic cells are usually positive for CD2, cytoplasmic CD3 (cCD3), CD56 and cytotoxic molecules (TIA1, perforin and granzyme B), occasionally express CD7 and CD30, whereas they are usually negative for surface CD3 (sCD3), CD4, CD8, CD5, CD16, CD57 and lack TCR gene rearrangement. The subset of cases of cytotoxic T-cell lineage (10–40% of cases) usually express sCD3, CD5, CD8 and show T-cell clonality. Nuclear expression of megakaryocyte-associated tyrosine kinase (MATK) is common.

EBER positivity represents a diagnostic criterion and a diagnosis of ENKTL should be considered unlikely if EBV is negative. The disease shows a type I latency pattern being often LMP1 negative. By gene expression profiling analysis, numerous oncogenic pathways have been identified to be activated in ENKTL-NT, including JAK/STAT and NF-kB [127,128] and several recurrent genetic and epigenetic (hypermethylation) alterations have been found in ENKTL-NT [139].

### 3.4. Differential Diagnosis

ENKTL-NT diagnosis can be challenging, particularly in cases that develop in extranasal sites, such as the GIT and if the pathologist is faced with small biopsy specimens. Tumors such as ENKTL-NT with superimposed conspicuous inflammatory infiltrate may be confused with inflammatory/infectious processes [126,140,141]. The differential diagnosis with CAEBV has been discussed in the paragraph dedicated to CAEBV.

The presence of an angiocentric and angioinvasive pattern may resemble lymphomatoid granulomatosis (LYG), a rare EBV-driven LPD, usually developing in the setting of immunodeficiency. [8,142,143]. LYG essentially affects the lungs and a diagnosis of LYG without lung involvement is questionable; additionally, GI involvement in LYG is rare. In LYG, the EBV-positive atypical large cells of B-cell origin are often small in number and dispersed among numerous reactive T lymphocytes. LYG shows angioinvasion by CD4-positive reactive T lymphocytes, whereas in cases of ENKTL-NT of T-cell origin, the neoplastic infiltrate is composed of CD8-positive cells.

Some EBV-negative lymphomas, which may occur in the GIT, such as indolent T-cell lymphoproliferative disorder (ITLPD), NK-cell enteropathy (NKCE), enteropathy-associated T-cell lymphoma (EATL), monomorphic epitheliotropic T-cell lymphoma (MEITL) have to be considered in the differential diagnosis [8,144,145]. The correct diagnosis of these entities requires attention to clinico-pathological features such as history of celiac disease, depth of lymphoid infiltrate, cytological and immunophenotypic features and association with EBV.

ITLPD of the GIT, included as a provisional entity in the current WHO classification, is a low grade clonal non-epitheliotropic LPD affecting almost any site of the GIT, particularly the small intestine and colon [8,144,145]. The clinical behavior is usually indolent with a chronic relapsing clinical course although cases of transformation to high grade lymphomas may occur. In ITLPD, small-sized and bland-looking T-lymphocytes involve mainly the lamina propria and sometimes extend to the muscolaris mucosae and submucosa; tumor masses and full thickness involvement are usually not present. The lymphocytes show a CD4-positive or CD8-positive phenotype, although double positive or double negative cases are seen. Characteristic findings of ENKTL-NT such as angioinvasion, angiocentricity and necrosis along with EBER and CD56 positivity are typically absent in ITLPD.

NKCE is a rare and indolent LPD of NK cells involving the GIT. Similarly to ITLPD, the infiltrate is usually superficial and not mass-forming. Necrosis and angioinvasion are absent; the cells of medium and large size express cCD3, CD2, CD7, CD56 and cytotoxic molecules and are EBER-negative. Pathologists need to be aware of this entity in order to avoid a misdiagnosis of ENKTL-NT.

MEITL and EATL are aggressive lymphomas, and, in both diseases, patients usually present with a mass lesion leading to intestinal obstruction and perforation with consequent acute symptoms. EATL is strongly associated with celiac disease and occurs mainly in the Western population; MEITL may occur worldwide although more frequently in Asian populations and is not linked with celiac disease.

EATL consists of large, pleomorphic cells and some histological features such as the often prominent inflammatory component along with extensive necrosis and angiocentric/angioinvasive growth may be suggestive of ENKTL-NT. In EATL, the cells usually express CD3, cytotoxic molecules and CD30 and are more often negative for CD4 and CD8; EATL is CD56 and EBER negative, although admixed EBER-positive by-stander small lymphocytes may be found.

MEITL consists of monomorphic and small to medium-sized cells with a striking epitheliotropism. In contrast to both ENKTL-NT and EATL, inflammatory background, necrosis and angioinvasion are absent. MEITL shows a distinctive phenotype, and is positive for CD3, CD8 and CD56 in the majority of cases, however, pathologists need to be aware of phenotypic variation as CD8 and CD56 may be negative and the aberrant expression of CD20 may lead to a misdiagnosis of MEITL as a B-cell lymphoma. Most MEITL are positive for MATK, a marker diagnostically helpful if expressed in >80% of cells; however, this marker alone is not useful in the differential diagnosis with ENKTL-NT, being commonly expressed even in ENKTL-NT. EBER is consistently negative in MEITL.

### 3.5. Treatment and Oucome

The prognosis of ENKTL-NT in its nasal form has recently improved due to more intensive therapeutic schemes. The extranasal form shows a worse outcome with poor response to therapy.

When localized, ENKTL-NT is treated with combined chemo-radiotherapy, whereas patients with disseminated disease are usually treated with L-asparaginase-based CT such as SMILE (dexamethasone, methotrexate, ifosfamide, L-asparaginase, etoposide) regimen [135]. After L-asparaginase-based therapy, relapse occurs in around 50% of patients with disseminated disease and, hence, there exists an urgent need for effective targeted therapy. Programmed cell death ligand 1 (PD-L1) expression has been observed in 50–80% of ENKTL-NT patients and checkpoint inhibitors (for instance pembrolizumab and nivolumab) have been demonstrated to be effective in some patients with advanced disease [135,146]. Additionally, CD38, a surface molecule expressed by ENKTL-NT, seems to be of potential value for target therapy [147]. Clinical trials evaluating JAK inhibition in ENKTL-NT are ongoing. It has been suggested that anti-CD38 antibodies or JAK inhibitors, combined with L-asparaginase-based CT, may represent a promising strategy to improve the prognosis of this aggressive lymphoma [148].

## 4. PTLDs

### 4.1. General Features and Etiology

PTLDs represent a potentially fatal complication, arising as a consequence of IS after both solid organ transplant (SOT) or HSCT [8,149,150,151,152,153]. PTLDs may occur at any time after transplant, but the first year post-transplant is the more critical.

PTLDs include a wide spectrum of diseases ranging from benign polyclonal LPDs, usually developing early post-transplant and regressing upon reduction of immunosuppression, to aggressive lymphomas requiring CT. The majority of PTLDs are of B-cell origin (85%) and over 80% are associated with EBV infection, whereas approximately 15% are of T-cell lineage and of these 30% are EBV-positive.

EBV plays a critical role in the development of most PTLDs [153]. After primary EBV infection occurring early in life, in immunocompetent individuals, the EBV genome is present in a latent form within B lymphocytes [1,2,3,4]. In conditions of IS, such as in the post-transplant setting, there is a reduction of T-cell function with a consequent lack of T-cell modulation on B-cell proliferation; this leads to uncontrolled proliferation of EBV-transformed B cells and, hence, to PTLDs. In EBV-positive PTLDs, either the infection is acquired after transplant in EBV-negative recipients or a latent EBV infection is reactivated because of IS.

Risk factors include EBV-negative recipients with EBV-positive donors, young age at transplant and more intensive immunosuppressive treatment. Seronegativity at the time of transplant increases the risk of PTLD occurrence by 10–75% [149,150,151,152,153]. SOT recipients aged <10 and >60 years are at increased risk of PTLD. Pediatric patients are at high risk, being often seronegative at the time of transplantation, whereas the high incidence of PTLDs at older age may be due to reduced immune surveillance. SOT recipients have higher risk (10%) of PTLDs than HSCT recipients (1–2%); PTLDs represent the most frequent post-transplantation malignancy in children and the second most common post-transplantation malignancy after skin tumor in adults.

The risk varies depending on the organ transplanted. Adult recipients of multi-organ, lung or intestinal transplants have a higher risk (=/>5%); those receiving liver and heart allografts have an intermediate risk (1–5%), whereas those receiving kidney transplant have a lower risk of PTLD development (<1%). This difference is in part due to the degree of immunosuppressive treatment administered for different SOT. Despite HSCT recipients having a lower risk of PTLD compared to SOT recipients, PTLDs following HSCT show a more aggressive course with high mortality [8,154]. The occurrence of PTLD-like lesions after autologous stem cell transplantation is rare and, according to the current WHO classification, should be included among iatrogenic immunodeficiency-associated LPDs rather than among PTLDs [8]. Patients receiving SOT require life-long immunosuppressive treatment and, therefore, may develop PTLDs even in a late phase, unlike patients receiving HSCT, who stop taking immunosuppressive agents after transplant and generally develop PTLDs within the first year.

Although PTLDs are mostly EBV-linked, approximately 20% of cases are EBV-negative. Early onset PTLDs are usually EBV-positive and may arise months after transplantation, whereas late onset PTLDs arising after years are more often EBV-negative. The pathogenesis of EBV-negative forms remains unclear, although various hypotheses including other infectious agents, chronic immune triggering by the graft and hit and run EBV infection have been proposed [155,156]. Some studies investigating the genomic profile of PTLDs revealed that EBV-negative cases show a genomic profile similar to that of DLBCL arising in immunocompetent individuals with a greater genomic complexity than EBV-positive PTLDs [157]. Despite these differences, similarly to EBV-positive forms, some EBV-negative PTLDs respond well to a reduction of immunosuppressive treatment.

PTLD clinical presentation is variable; PTLDs may be incidentally found due to the paucity of symptoms; sometimes non-specific symptoms such as pyrexia and malaise are present, whereas in other cases the disease is fulminant with multi-organ failure. Almost any organ may be involved, although lymph nodes (33%), GIT (29%), central nervous system (CNS) (13%), liver (12%) and lung (4%) are the sites most commonly affected [151]. In PTLDs occurring after SOT, PTLD may involve the transplanted organ, with allograft dysfunction and, therefore, the correct diagnosis may be difficult as infection and allograft rejection may cause similar clinical manifestations.

### 4.2. PTLDs and GIT

The GIT is one of the sites that is most frequently involved by PTLDs, particularly in kidney transplants [150,158]. Isolated GI involvement has been reported in both the pediatric population and in adults [150,159]. Isolated GI involvement was found in 19% of 181 adult French renal transplant recipients [151]. Symptoms may be nonspecific and rather vague. In a large series by Cruz et al. it was noted that GI PTLDs were often of late onset, frequently involved the lower GIT (small bowel and right colon 81%) and were more often monoclonal [160]. Intestinal obstruction represents a common indication for surgery, whereas GI bleeding and perforation are associated with a worse outcome [160].

### 4.3. Histology, Immunophenotype and Genetic Profile

Based on the criteria defined in the current WHO classification, PTLDs are sub-classified into four categories: (1) Non-destructive PTLDs including plasmacytic hyperplasia (PH), florid follicular hyperplasia and IM-like PTLD; (2) Polymorphic PTLD (P-PTLD); (3) Monomorphic PTLD (M-PTLD), further sub-divided in B-cell, T-cell, NK-cell types and (4) cHL-like PTLD [8] (Table 2).

Non-destructive PTLDs tend to occur at a younger age and are more frequent in individuals with no prior EBV exposure. They are by definition characterized by their preservation of underlying tissue architecture. Non-destructive PTLDs are mass-forming lesions. Lymph nodes, tonsils and adenoids are more frequently involved than other extranodal sites. PH shows plasma cells (PCs) with scattered immunoblasts (IBs); whereas IM-like lesions predominantly show IBs and occasionally Reed-Sternberg (RS)-like cells and PCs; the presence of necrosis and high mitotic activity may suggest malignancy. The histology is non-specific, so the diagnosis can be made on the basis of transplant history and the presence of EBV positivity. Non-destructive PTLDs are usually polyclonal, although oligoclonality may be detected; an analysis of episomal EBV DNA demonstrates that the virus is also polyclonal or oligoclonal.

P-PTLD represents the most common subtype in children and is mostly related to primary EBV infection. It consists of an heterogeneous population of small and medium-sized lymphocytes, PCs and IBs effacing the tissue architecture. Areas of geographical necrosis and cells resembling Reed Sternberg cells (RSCs) are frequently present. P-PTLDs do not fulfil the diagnostic criteria for any of the lymphoma types recognized in immunocompetent individuals. In P-PTLD there are numerous, variably-sized CD20-positive cells admixed with many predominantly small-sized T-cells. Many EBER-positive cells are present as well as numerous CD30-positive elements. However, in contrast to cHL, the CD30-positive RSCs express CD20 and lack CD15. P-PTLD are mostly monoclonal, although the clones are less predominant than in M-PTLD.

M-PTLDs are monoclonal proliferations fulfilling the diagnostic criteria for either B-cell or T/NK-cell neoplasms, recognized in immunocompetent hosts. The vast majority of M-PTLDs are of B-cell lineage and they resemble, both clinically and histologically, either DLBCL or, less frequently, Burkitt lymphoma (BL) or a plasma cell tumor. The small B-cell lymphomas arising in the setting of transplantation are not classified among PTLDs with the exclusion of the EBV-positive extranodal marginal zone lymphoma of mucosa-associated lymphoid tissue, arising in the skin and subcutaneous tissue [8,161]. The EBV-positive DLBCL, which represents the largest part of M-PTLDs, often shows a non-germinal center (GC) phenotype, whereas EBV-negative cases more often present a GC phenotype.

Molecular studies reveal monoclonal IGH gene rearrangements and that the EBV genetic material is also monoclonal. T-cell clonality may be detected in the M-PTLDs of B-cell type. Cytogenetic abnormalities are common in monomorphic B-cell PTLDs (72%) compared with P-PTLDs, showing cytogenetic abnormalities in only 15% of cases. The most frequent clonal abnormalities in M-PTLDs are trisomies 9 and/or 11 followed by rearrangements of 8q24.1, 3q27 and 14q32 [8,162].

M-PTLDs of T/NK cell origin represent around 15% of all PTLDs. Any WHO type of T/NK cell lymphomas arising in non-transplanted patients may be seen in the post-transplant setting. The majority of T-cell PTLDs are EBV negative, whereas PTLDs of NK cell origin are mainly EBV positive with EBV clonality [152]. T-cell clonality is present in M-PTLDs of T-cell origin.

c-HL PTLD is a rare form of PTLD, occurring mainly after renal transplant and is a late complication of transplantation. It is often of a mixed cellularity type and mostly EBV positive. The diagnosis of cHL PTLD should be performed in the presence of a typical morphology and phenotype; in particular CD30 and CD15 should be co-expressed [8,163]. Caution is advisable as RS-like cells are also present in other types of PTLDs; however, in these cases, RS-like cells are usually positive for CD45 and CD20 and negative for CD15 [8,150,163].

PTLDs commonly demonstrate a type III latency pattern, although more restricted latency patterns are found.

### 4.4. Differential Diagnosis

Although a biopsy is required for PTLD diagnosis, a rise in EBV-viral titers should be considered highly suspicious for EBV-associated PTLD [164]. In any case, a complete history needs to be provided with the specimen, as a careful clinicopathological correlation is essential for PTLD diagnosis. A clear separation among the different PTLD categories is not always possible as they are likely to represent a pathological spectrum.

The histology, in particular of non-destructive PTLDs, is non-specific and a full patient’s history is essential.

PH may simulate a plasma cell neoplasm, but PH is polyclonal. IM-like PTLD, especially in cases with necrosis and high mitotic index, may resemble EBV-positive DLBCL, NOS. Unlike EBV-positive DLBCL, NOS, IM-like PTLD retains, at least in part, the tissue architecture, blasts are of both B-cell and T-cell lineages and B-cell clonality is negative.

RS-like cells are often seen in IM-like PTLD and in P-PTLD; therefore, cHL needs to be excluded, as mentioned above.

A graft reaction may show a polymorphic lymphoid infiltrate; the presence of numerous EBV positive cells favors P-PTLD diagnosis.

Similarly to the corresponding T-cell lymphomas occurring in non-transplanted patients, in M-PTLD of T-cell lineage, the aberrant expression of B-cell markers by neoplastic T-cells may erroneously lead to a diagnosis of M-PTLD of B-cell lineage; cases positive for CD30 and EBV may simulate cHL-PTLD.

**Figure 1:**
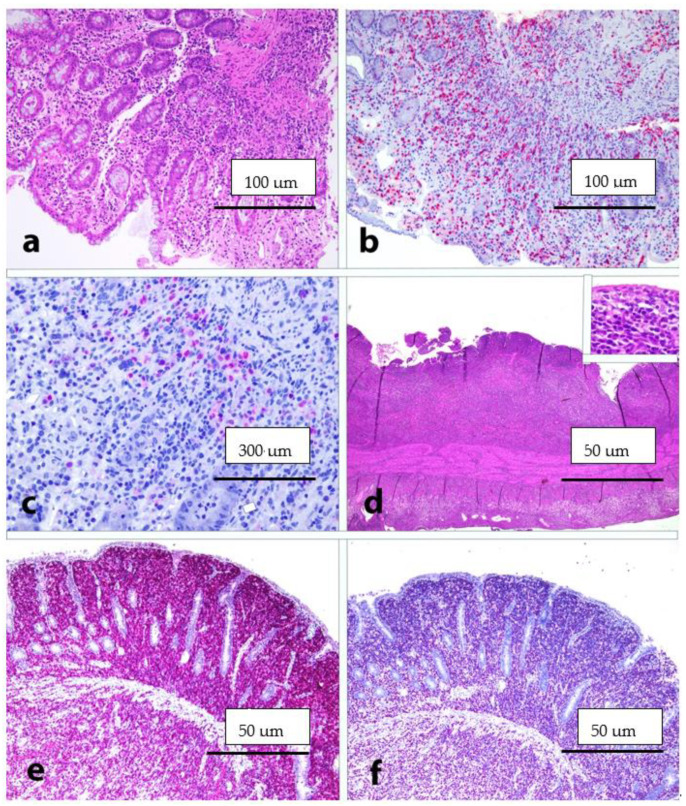
(**a**) PTLD: Medium power view showing a mucosal infiltrate with preserved tissue architecture (HE, 20× magnification); (**b**) PTLD: CD3 staining highlighting the mucosal infiltrate (immunohistochemical staining, 20×); (**c**) PTLD: EBER positivity (in situ hybridization; 40× magnification); (**d**) ENKTL, nasal-type: low power view showing the neoplastic infiltrate diffusely involving the intestinal wall; in the inset: details of neoplastic cells (HE, 10× magnification; inset, 40× magnification); (**e**) ENKTL, nasal-type: CD56 expression (immunohistochemical staining, 10× magnification); (**f**) ENKTL, nasal-type: EBER positivity (in situ hybridization; 10× magnification). All images are originals from Prof. S.A.P.

Histological and immunophenotypic details of ENKTL nasal-type and polymorphic PTLD of the GIT are presented in Figure 1.

### 4.5. Treatment and Outcome

A reduction of immunosuppression (RI) represents the mainstay of PTLD treatment.

Non-destructive PTLDs usually regress with RI and the outcome is good, unless graft rejection develops [165].

P-PTLDs and a minority of M-PTLDs may regress with RI. The main concerns with RI are the risk of graft rejection and the time necessary for an initial response (approximately 3–5 weeks). EBV-positive PTLDs are more responsive to RI than EBV-negative cases [166].

Unresponsiveness to RI is more common in patients over 50 years, late-onset PTLD, bulky disease, multiorgan involvement, high lactate dehydrogenase and patients with an advanced stage of the disease [151,166]. In some P-PTLDs and most M-PDLDs of B-cell lineage not responding to RI, Rituximab treatment is sometimes indicated in association with CT [167,168]. Different chemotherapeutic schemes have been used in the treatment of M-PTLD, depending on the type of lymphoma, with CHOP being the most commonly used scheme [168]. Complete remission with combination CT is achieved in more than 90% of cases; unfortunately treatment-related toxicity remains high with an associated mortality of over 50% [169]. Local therapies such as RT and surgery may be used in localized PTLDs, usually in combination with RI. T-cell immunotherapy represents another therapeutic strategy [170]. Unmanipulated donor lymphocytes represent a T-cell immunotherapy option as well as donor derived EBV-transformed lymphoblastoid cell line, and off-the-shelf EBV-specific T cells [171,172,173].

## 5. Conclusions

EBV-driven LPDs represent a spectrum of entities with variable clinical behaviors. An accurate diagnosis begins from the awareness of this group of disorders by clinicians and pathologists and requires a combination of clinical, histological, immunophenotypic and molecular features. In GIT, the diagnosis can be even more complex as pathologists are often faced with small biopsy specimens. Additionally, EBV-associated T/NK-cell LPDs represent a particularly challenging diagnostic setting, owing to the discrepancy between the often aggressive clinical course and the subtle histological features which may be easily overlooked, leading to a misdiagnoses of these disorders as benign and inflammatory processes, ultimately resulting in improper treatments.

## Figures and Tables

**Table 1:** Lymphoproliferative disorders of T/NK-cell origin with GIT involvement.

Variation	ENKTL-NT	CAEBV	EATL	MEITL	ITLPD
Sites of GIT (in order of frequency)	Small bowel Large bowel Stomach Esophagus	Large bowel Small bowel Stomach	Small bowel; Large bowel Stomach	Small bowel; large bowel; stomach	All sites of GIT; Small bowel or large bowel (more often)
Macroscopic features	Mucosal ulcer or ulcerating mass	Ulcer	Ulcerating lesion or stricture	Tumor mass	Subtle features: hyperaemic mucosa; nodularity; Prominent folds; polyps
Coeliac disease	No	No	Yes	No	No
Histology	Cells of variable size; Admixed inflammation; Necrosis; Angioinvasive/angiocentric pattern	Infiltrate within mucosa, rarely submucosa or muscle layer involvement; Small/medium-sized cells often with mild atypia	Medium/ large- sized cells; Admixed inflammation; Necrosis; Angioinvasive/ angiocentric pattern	Monomorphic Medium- Sized cells; Epitheliotropism (often)	Non-destructive mucosal infiltrate (rarely muscolaris mucosae or submucosa involved)
Cell of origin	NK-cell often; T-cell	T-cell (59%); NK-cell (41%); B-cell rarely	T-cell	T-cell	T-cell
IHC	NK cell origin: CD2+ cCD3+ CD56- cytotoxic molecules+ CD7+/− CD30+/− sCD3−, CD4− CD8− CD5− CD16− CD57− MATK+. T-cell origin: sCD3+ CD5+ CD8+ MATK+	T-cell origin: CD4 > CD8	CD3+ CD7+ CD103+ CD5− CD4− CD8− often cytotoxic molecules+ CD30+ (often) TCRB+ (in some cases) TCRG+ (in some cases)	CD3+ CD8+ CD56+ CD5− TCRG+ (often) TIA1+ CD20 aberrant+ (20% of cases) MATK+	CD3+ CD8+ often; CD4+ some cases; CD4− CD8− rarely; CD2+ CD5+ CD7+/− TIA1+ Granzyme B− TCR alfa beta+ CD56-
EBER-ISH	Positive	Positive	Negative	Negative	Negative
Proliferative fraction	High	Low	High	High	Low
T-cell clonality	T-cell clonality in cases of T-cell origin	T-cell clonality; more rarely oligoclonality or polyclonality	T-cell clonality	T-cell clonality	
Clinical course	Aggressive	Variable (NK forms: more favorable course)	Aggressive	Aggressive	Indolent

CAEBV: chronic active Epstein–Barr virus infection; EBER-ISH: EBV-encoded RNA in situ hybridization; EATL: enteropathy-associated T-cell lymphoma; ENKTL-NT: extranodal NK/T-cell lymphoma, nasal type; gi-ITLPD: indolent T-cell lymphoproliferative disorder of the gastrointestinal tract; GIT: gastrointestinal tract; IHC: immunohistochemistry; MATK: megakaryocyte-associated tyrosine kinase; MEITL: monomorphic epitheliotropic intestinal T-cell lymphoma; TCRB: T-cell receptor beta gene rearrangement; TCRG: T-cell receptor gamma gene rearrangement.

**Table 2:** Characteristics of PTLDs.

WHO Classification	EBV Association	Time of Post-Transplant Onset	Clonality
Non-destructive PTLD	Often EBV-positive	Often early	Often polyclonal (oligoclonality may be present)
Polymorphic PTLD	Often EBV-positive	Often early	Often monoclonal
Monomorphic PTLD (B-cell origin)	EBV-positive and EBV-negative	Both early and late	B-cell clonality present; sometimes T-cell clonality
Monomorphic PTLD (T/NK-cell origin)	T-cell origin: often EBV negative; NK-cell origin: often EBV-positive	Often late	T-cell clonality in PTLDs of T-cell origin
cHL-PTLD	Often EBV-positive	Often late	Clonality rarely detected

EBV: cHL: classic Hodgkin lymphoma; Epstein–Barr virus; PTLD: post-transplant lymphoproliferative disorder; WHO: World Health Organization.
